# Involvement of the PI3K/AKT Intracellular Signaling Pathway in the AntiCancer Activity of Hydroxytyrosol, a Polyphenol from *Olea europaea*, in Hematological Cells and Implication of HSP60 Levels in Its Anti-Inflammatory Activity †

**DOI:** 10.3390/ijms23137053

**Published:** 2022-06-24

**Authors:** Alberto M. Parra-Perez, Amalia Pérez-Jiménez, Isabel Gris-Cárdenas, Gloria C. Bonel-Pérez, Luis M. Carrasco-Díaz, Khalida Mokhtari, Leticia García-Salguero, José A. Lupiáñez, Eva E. Rufino-Palomares

**Affiliations:** 1 Department of Biochemistry and Molecular Biology I, Faculty of Sciences, University of Granada, Avenida Fuentenueva, 1, 18071 Granada, Spain; alberto.parra@genyo.es (A.M.P.-P.); igris@cicbiogune.es (I.G.-C.); gloriacbonel@gmail.com (G.C.B.-P.); luismiguelcarrascodiaz@gmail.com (L.M.C.-D.); khalidafadoua1@gmail.com (K.M.); elgarcia@ugr.es (L.G.-S.); 2 GENYO, Centre for Genomics and Oncological Research: Pfizer, University of Granada, Avenida de la Ilustración, 114, 18016 Granada, Spain; 3 Department of Zoology, Faculty of Sciences, University of Granada, Avenida Fuentenueva, 1, 18071 Granada, Spain; calaya@ugr.es; 4 CIC bioGUNE, Centre for Cooperative Research in Biosciences, 48160 Derio, Spain; 5 CNIO, Spanish National Cancer Research Centre, Calle Melchor Fernández Almagro, 3, 28029 Madrid, Spain; 6 Sant Pau Hospital Research Institute Foundation, Autonomous University of Barcelona (UAB), 08193 Barcelona, Spain; 7 Laboratory of Bioresources, Biotechnologies, Ethnopharmacology and Health, Department of Biology, Faculty of Sciences, Mohammed I University, Oujda 60000, Morocco

**Keywords:** apoptosis, cell cycle arrest, HSP60, hydroxytyrosol, inflammation, leukemia cells, PI3K/AKT signaling pathway, ROS level

## Abstract

Hydroxytyrosol (HT), the main representative of polyphenols of olive oil, has been described as one of the most powerful natural antioxidants, also showing anti-inflammatory, antimicrobial, cardioprotective and anticancer activity in different type of cancers, but has been little studied in hematological neoplasms. The objective of this work was to evaluate the anticancer potential of HT in acute human leukemia T cells (Jurkat and HL60) and the anti-inflammatory potential in murine macrophages (Raw264.7). For this, cytotoxicity tests were performed for HT, showing IC_50_ values, at 24 h, for Jurkat, HL60 and Raw264.7 cells, of 27.3 µg·mL^−1^, 109.8 µg·mL^−1^ and 45.7 µg·mL^−1^, respectively. At the same time, HT caused cell arrest in G_0_/G_1_ phase in both Jurkat and HL60 cells by increasing G_0_/G_1_ phase and significantly decreasing S phase. Apoptosis and cell cycle assays revealed an antiproliferative effect of HT, decreasing the percentage of dividing cells and increasing apoptosis. Furthermore, HT inhibited the PI3K signaling pathway and, consequently, the MAPK pathway was activated. Inflammation tests revealed that HT acts as an anti-inflammatory agent, reducing NO levels in Raw264.7 cells previously stimulated by lipopolysaccharide (LPS). These processes were confirmed by the changes in the expression of the main markers of inflammation and cancer. In conclusion, HT has an anticancer and anti-inflammatory effect in the cell lines studied, which were Raw264.7, Jurkat, and HL60, and could be used as a natural drug in the treatment of liquid cancers, leukemias, myelomas and lymphomas.

## 1. Introduction

Cancer is one of the leading causes of mortality worldwide, especially in developed countries due to the aging of the population, according to the World Health Organization (WHO) in 2018 [1]. Malignant hematologic neoplasms account for about 10% of cancer cases diagnosed per year. These cancers include leukemias, lymphomas and myelomas, in which normal hematopoiesis does not occur and they can arise during any stage of the blood cell development, impairing their production and function [2].

T-cell acute lymphoblastic leukemia is an aggressive hematologic malignancy arising from the rapid proliferation of T-cell progenitors. It represents 15% of pediatric cases and 25% of adults, and it has a 5-year survival rate below 10% [3]. Their treatment is based on combination chemotherapy, hematopoietic stem cell transplantation, immunotherapy and directed treatment at molecular targets [4]. However, the side effects of the treatments, the high mortality rate, the late diagnosis and the increase in new cases, have directed scientific efforts towards uncovering knowledge on the molecular mechanisms that underlie this disease in order to develop new preventive and targeted therapies. Acute promyelocytic leukemia represents 3% of leukemias and it has a higher prevalence in adults. It is produced by the generation of undifferentiated blood cells [5].

In malignant cells, the cell cycle regulatory mechanism is altered mainly due to the overexpression of certain cyclins. If the cell cycle were arrested or apoptosis were induced, their proliferation would be prevented and cancer progression would be delayed [6,7]. Moreover, inflammation induced by tumor modifies this microenvironment [8]. Therefore, the use of natural products that are involved in these processes could help to prevent cancer disease.

The Mediterranean diet has been widely studied for its association with a better state of health, since it provides high levels of antioxidants derived from the intake of fruits, vegetables and olive oil. Olive oil is extracted from the fruit of the olive tree (*Olea europaea* L.) and is associated with the prevention of human pathologies such as cancer, cardiovascular diseases and diabetes, among others [9]. It is mainly composed of triglycerides (98–99%), being the most abundant monounsaturated oleic acid. In addition, it presents a great diversity of terpenic acids and phenolic compounds, such as phytosterols and tocopherols, both in the leaf and in the fruit, related to health benefits.

**Figure 1:**
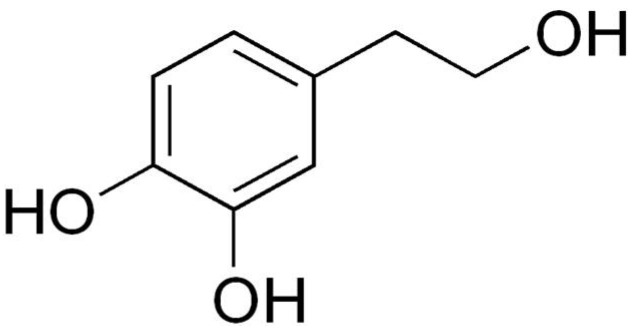
Structure of Hydroxytyrosol, 3,4-dihydroxyphenylethanol (DOPET). IUPAC name: 4-(2-Hydroxyethyl)-1,2-benzenediol.

In this sense, different research groups have studied the biological effects of some of its components, most of them related to cell growth processes. Among these effects, their positive impact has been demonstrated during cell growth under normal conditions [10,11,12,13]. Under pathological cell growth, they present important anticancer [14,15,16,17,18,19,20], antiproliferative, anti-inflammatory [21,22,23] and antioxidants effects [24,25,26]. Additionally, they induce changes in the expression of cytoskeletal proteins [27], in transcriptomic and metabolomic reprogramming [28] and in cell differentiation under metabolic syndrome conditions [29]. Among the phenolic compounds, we highlight the oleuropein-derived polyphenols [30]. They constitute one of the most numerous, varied and ubiquitous groups of secondary metabolites in plants and they have anti-inflammatory and antimicrobial activities [31]. Among the most important polyphenols, due to their abundance in olive oil, we find hydroxytyrosol (HT, 3,4-dihydroxyphenylethanol), an amphipathic phenolic compound with a molecular weight of 154.16 g·mol^−1^ (Figure 1).

HT in oil is found in its free form, as acetate or as part of more complex compounds such as oleuropein or verbascoside [9]. Due to its polar nature, HT is found in large quantities in oil processing residues, such as pomace oil, “alpeorujo” and “alpechín”. In fact, the by-products of olive oil production constitute the main source of HT [32].

HT bioactivities are a direct consequence of its structure and the catechol group (benzene ring with two alcohol groups). This molecule acts as an antioxidant by capturing the superoxide anion, generated in cells in different ways and protecting against oxidative damage; and as a metal chelator. It also inhibits proliferation and induces apoptosis in cancer cells, prevents DNA damage and has anti-inflammatory and antithrombotic activity, among many other properties [9,33,34].

Several studies describe the HT anticancer properties in different tumor cell lines, such as colon, breast, liver and prostate [34,35,36,37,38] and anti-inflammatory activity was demonstrated in monocytic leukemia [39,40].

Due to the promising results shown by studies on the anticancer potential of HT in solid cancer, the purpose of this work was to evaluate the said anticancer capacity in hematological malignancies, such as cell lines of T-cell acute lymphoblastic leukemia and acute promyelocytic leukemia (Jurkat and HL60). Both are liquid tumors, which were not considered in previous HT research for possible prevention or treatment. In addition, anti-the inflammatory capacity is evaluated in the murine macrophage cell line (Raw264.7), suggesting that it could be regarded as a potential source of natural anti-inflammatory agents.

## 2. Results

### 2.1. Hydroxytyrosol Has a Cytotoxic Effect on Different Blood Cell Lines

**Figure 2:**
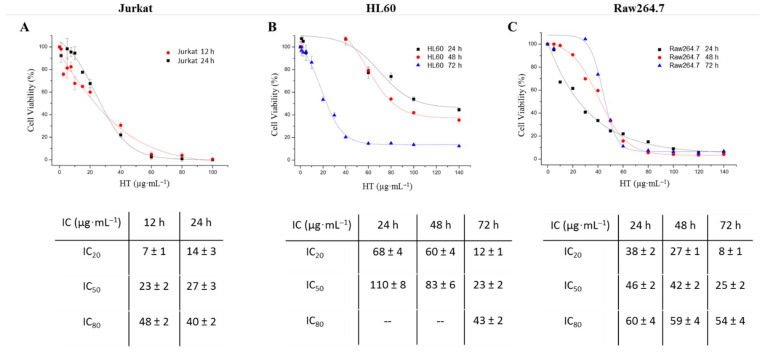
Cytotoxicity curves of hydroxytyrosol during 12 and 24 h in Jurkat cells (**A**); 12, 48 and 72 h in HL60 (**B**) and Raw264.7 at the same three times (**C**). The assays were performed using three independent populations of cells. Results expressed as μg·mL^−1^ are mean ± SEM. IC_80_, IC_50_ and IC_20_ values for the Jurkat, HL60 and Raw264.7 lines after 12, 24, 48 and 72 h of incubation with hydroxytyrosol and are indicated in the tables below the graph. These results were obtained by a dose–response model using OriginPro 8 software (OriginLab^®^, Northampton, MA, USA). IC, inhibitory concentration.

Hydroxytyrosol’s effect was evaluated using the MTT assay on the Raw254.7 and resazurin assay on Jurkat and HL60 cell lines. The viability results obtained were represented in percentages (%) with respect to the different concentrations of compound administered (in μg·mL^−1^) for each cell line at different times of exposure, showing a sigmoidal adjustment (Figure 2). Similarly, the required HT doses to decrease cell viability by 20%, 50% and 80% (IC_20_, IC_50_ and IC_80_) were calculated.

In Jurkat cells (Figure 2A), cell viability showed a dose-dependent behavior, showing a clear sigmoidal profile, although being little affected by time. As a consequence, the IC_50_ values obtained at 12 and 24 h were not significantly different (23.26 ± 1.85 and 27.32 ± 2.92 µg-mL^−1^, respectively, *p* = 0.31), as was the case for the IC_20_ values (6.89 ± 2.05 and 14.4 ± 3.86 µg-mL^−1^, *p* > 0.16). A similar behavior was observed for the HT values reaching 80% cell death (IC_80_), at 12 and 24 h, where neither presented significant changes (*p* = 0.06), these values being 47.65 ± 2.04 and 40.25 ± 1.98 µg-mL^−1^, respectively.

Figure 2B shows that, for the HL60 line, HT reduced cell viability in a dose- and time-dependent way, as the IC_50_ and IC_20_ values decreased with this treatment in a progressive manner for the three times studied (24 h, 48 h and 72 h). The IC_80_ values at 24 and 48 h could not be calculated since the model-estimated concentration exceeded the maximum concentration used in the assay (140 μg·mL^−1^). The IC_80_, IC_50_ and IC_20_ values obtained at 72 h were, respectively, 42.85 ± 2.14, 23.3 ± 1.80 and 11.48 ± 0.91 μg·mL^−1^.

Finally, the values of IC_20_, IC_50_ and IC_80_ in Raw264.7 presented a dose and time dependent behavior such that the cell viability was affected by the HT concentration and the incubation time of the same. At 24 h, the values of IC_20_, IC_50_ e IC_80_ were 8.22 ± 0.65, 24.79 ± 1.90 and 60.39 ± 3.88 μg·mL^−1^, respectively. However, after 48 h, the behavior of the compound ceased to be time dependent and the dose required to achieve both 50% (IC_50_) and 20 % (IC_80_) cell viability was equal at 48 and 72 h, of 42.09 ± 2.04 and 45.73 ± 2.20 μg mL^−1^ and 58.49 ± 3.98 and 54.08 ± 3.91 μg mL^−1^, respectively (Figure 2C).

### 2.2. Hydroxytyrosol Induces G_0_/G_1_ Arrest in Jurkat and HL60 Cells

**Figure 3:**
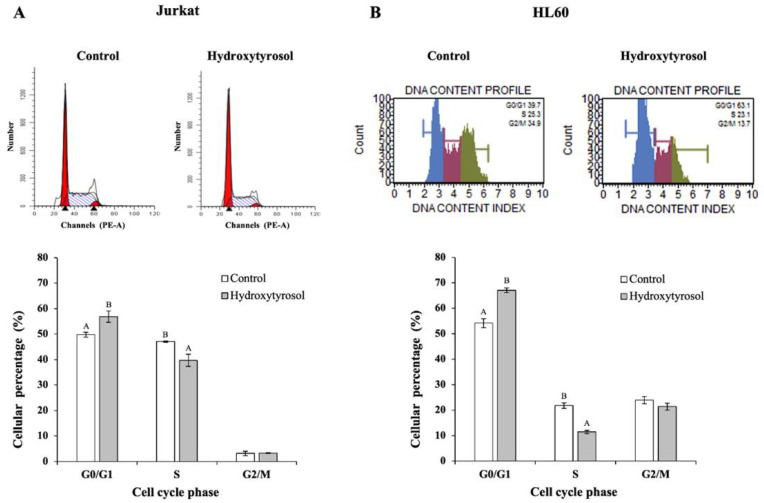
Effect of hydroxytyrosol on the cell cycle in Jurkat (panel **A**) and HL60 cell lines (panel **B**). Cell cycle analysis was performed with flow cytometry. Panel A corresponds to Jurkat cells and panel B to HL60 cells. Cells were treated with hydroxytyrosol IC_50_ for 24 h for Jurkat and 72 h for HL60 cells. Histograms from a representative experiment showing the effects of hydroxytyrosol on the cell cycle profile are represented at the top (Jurkat panel A and HL60 panel B). The lower part represents the percentage of cells in each of the phases of the cell cycle. Values are expressed as mean ± SEM (*n* = 3). Different capital letters indicate significant differences between treatments for each phase of the cell cycle (*p* < 0.01).

For the purpose of analyzing the possible effect of HT in the cell cycle for each line studied, the proportion of cells that were in each phase of the cycle (G_0_/G_1_, S and G_2_/M) were measured in control and HT conditions using the flow cytometer. Figure 3 shows the histograms and the plots of the cell cycle assay generated.

The results of the study of the cell cycle in Jurkat showed that HT caused a significant increase of 7% in the number of cells that are in the G_0_/G_1_ phase with respect to the control, as well as a significant decrease in the S phase (Figure 3A).

However, no significant changes were observed in the G_2_/M phase. This indicates that HT led to cell arrest in the G_0_/G_1_ phase. Similar results were found in HL60 since HT produced significant cell arrest in phase G_0_/G_1_ (Figure 3B), with an increase of 13% in cells in this phase against control condition.

### 2.3. Hydroxytyrosol Enhances Apoptosis in Jurkat and HL60 Cells

**Figure 4:**
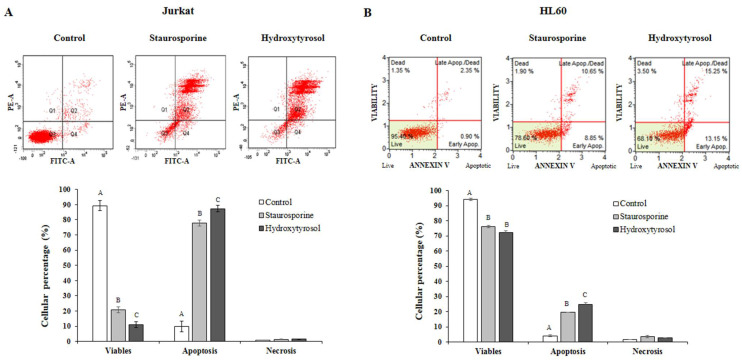
Effect of hydroxytyrosol on cell apoptosis levels in Jurkat (panel **A**) and HL60 (panel **B**) cell lines. Apoptosis values were obtained using flow cytometry. A positive control in the presence of staurosporine (1 μg·mL^−1^) was used. Panel A corresponds to Jurkat cells and panel B to HL60 cells. Cells were treated with hydroxytyrosol IC_50_ for 24 h for Jurkat and 72 h for HL60 cells. Top: Fluorescence diagrams from a representative experiment are depicted and show the effect of hydroxytyrosol on different cell states, viability, early apoptosis, late apoptosis, and necrosis (Jurkat panel A and HL60 panel B). Below: the percentage of cells in each of these states are represented. Values are expressed as mean ± SEM (*n* = 3). Different capital letters indicate the existence of significant differences (*p* < 0.01). Quadrants: Q1, necrosis; Q2, late apoptosis; Q3, living cells; Q4, early apoptosis.

The apoptosis assay provides information on the type of cell death (apoptosis and necrosis) that occurs in each line and for each situation studied, including the negative control, positive control (Staurosporine) and treatment with HT. Figure 4 shows the diagrams and graphs of the apoptosis assay generated by the flow cytometer during the analysis for each experimental condition.

In Jurkat cells, the negative control showed a higher percentage of viable cells (89.3%) compared to apoptotic (9.8%) and necrotic cells (0.9%). The presence of staurosporine (positive control) presented a cell viability percentage of 22.3% and an increase of 76.1% in cells in apoptosis, while the addition of HT produced a significant increase in apoptosis (87.3%) and a decrease (*p* < 0.001) in the percentage of cell survival (11.1%) (Figure 4A).

With reference to the HL60 line, a similar pattern was observed as that found in Jurkat cells. Cell viability and apoptosis for the negative control were 94.2% and 4.2%, respectively. Both the presence of staurosporine and HT caused a significant decrease in-viable cells (77.0% and 72.1%, respectively) as well as a significant increase in the degree of apoptosis of 19.6% and 25.0%, respectively (Figure 4B). In none of the situations studied were significant changes generated for the rate of necrotic cells, which were low, never exceeding 3.5%. Therefore, HT exerts a clear pro-apoptotic effect in both lines.

### 2.4. Hydroxytyrosol Increases ROS Production in Jurkat and HL60 Cells

**Figure 5:**
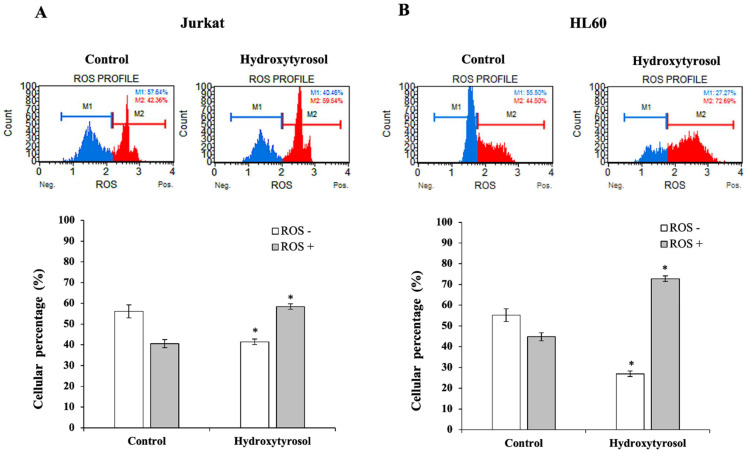
Effect of hydroxytyrosol on the levels of Reactive Oxygen Species (ROS) production in Jurkat (panel **A**) and HL60 (panel **B**) cell lines. The percentage of negative ROS, ROS (−), and positive ROS, ROS (+), values observed for each treatment are represented. Panel A corresponds to Jurkat cells and Panel B to HL60 cells. Cells were treated with hydroxytyrosol IC_50_ for 24 h for Jurkat (A) and 72 h for HL60 (B) cells. Histograms from a representative experiment showing the effects of hydroxytyrosol on ROS production are plotted at the top. The lower part represents the percentages of ROS (+) and ROS (−) in the control and treated cultures. Values are expressed as mean ± SEM (*n* = 12). The presence of an asterisk indicates significant differences (*p* < 0.001) between the levels of ROS (+) and ROS (−) versus control and treatment.

Intracellular Reactive Oxygen Species (ROS) levels, a marker of cellular oxidative stress, were measured by FACS both in the absence and in the presence of HT. Figure 5 includes bar graphs with the percentage of cells in each situation studied with the detection of ROS, ROS (+) or without the detection of ROS (−) in both blood cancer cell lines, Jurkat and HL60.

In the case of Jurkat cells (Figure 5A), at 24 h, the presence of HT led to 58.4% of cells identified as ROS (+) compared to 40.5% of the untreated group (*p* < 0.05). This compound caused a significant decrease in ROS (−) levels of 27.6% and an increase of 50.5% in ROS (+). Similar results were found with HL60 cells (Figure 5B) in which HT treatment produced significant changes, with a decrease of 55.2% in ROS (−) cells and an increase of 58.7% in the cell number identified as ROS (+).

These results clearly show that the treatment with HT of Jurkat and HL60 cells significantly increases the oxidative stress of these cells and therefore facilitates the pro-apoptotic activity of this polyphenol.

### 2.5. Hydroxytyrosol Produces up and Down-Regulation of Target Proteins of Apoptosis and Proliferation Processes

**Figure 6:**
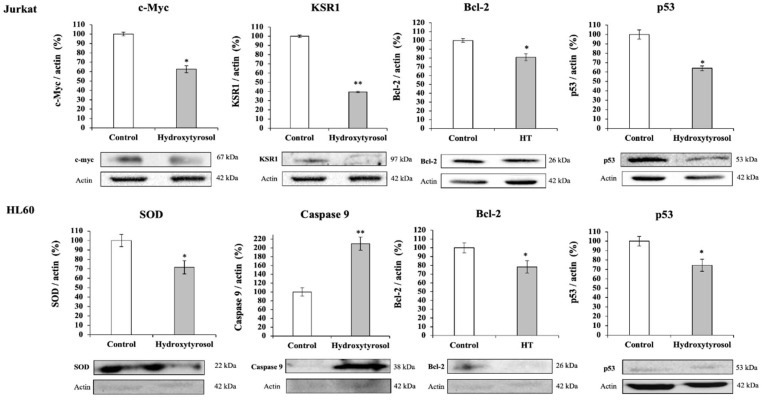
Effect of hydroxytyrosol (HT) on the expression levels of different marker proteins for cell proliferation and differentiation (c-Myc, KSR1), apoptosis (Bcl-2, p53, Caspase 9) and cellular oxidative stress (SOD) in Jurkat and HL60 cell lines controls and exposed to the IC_50_ concentration of HT for 24 h in Jurkat cells and 72 h in HL60 cells. Quantification of protein levels by densitometric analysis is shown in bar graphs. Results are means ± SEM (*n* = 9) and are expressed as percentage of expression compared to actin levels in Western-blot analysis. The presence of the asterisk indicates significant differences between the control and treated cells (* *p* < 0.01; ** *p* < 0.001). KSR1, Kinase suppressor of Ras 1; Bcl2, Anti-apoptotic B-cell lymphoma 2; SOD, Superoxide dismutase.

The protein expression of different markers of the apoptosis, stress and cell proliferation pathways (c-Myc, encoded proteins for cell proliferation and differentiation, KSR1, Ras kinase suppressor protein, Bcl-2, anti-apoptotic B-cell lymphoma-2 protein and non-phosphorylated tumor protein, p53, Superoxide dismutase related with antioxidant defense, SOD and the initiator protein of apoptosis, Caspase 9) was measured using Western blot. The level of expression obtained for each protein of interest was normalized according to actin (constitutive protein) and subsequently referred to that obtained in the control situation (Figure 6).

The results obtained show that HT causes a significant decrease in cell differentiation in Jurkat cells by decreasing the expression levels of cMyc and KSR1 together with a significant increase in cell apoptosis by decreasing the levels of Bcl2 and Phospho-p53. As for HL60 cells, hydroxytyrosol administration resulted in increased cell apoptosis as a consequence of increased caspase-9 levels and a significant decrease in Bcl2 and phospho-p53, together with an effective antioxidant defense by modifying SOD levels. All this indicates a clear anticancer effect of HT in both cell lines of hematological cancers.

### 2.6. Hydroxytyrosol Modulates Dual PI3K/MAPK Signaling Pathway in Jurkat Cells

**Figure 7:**
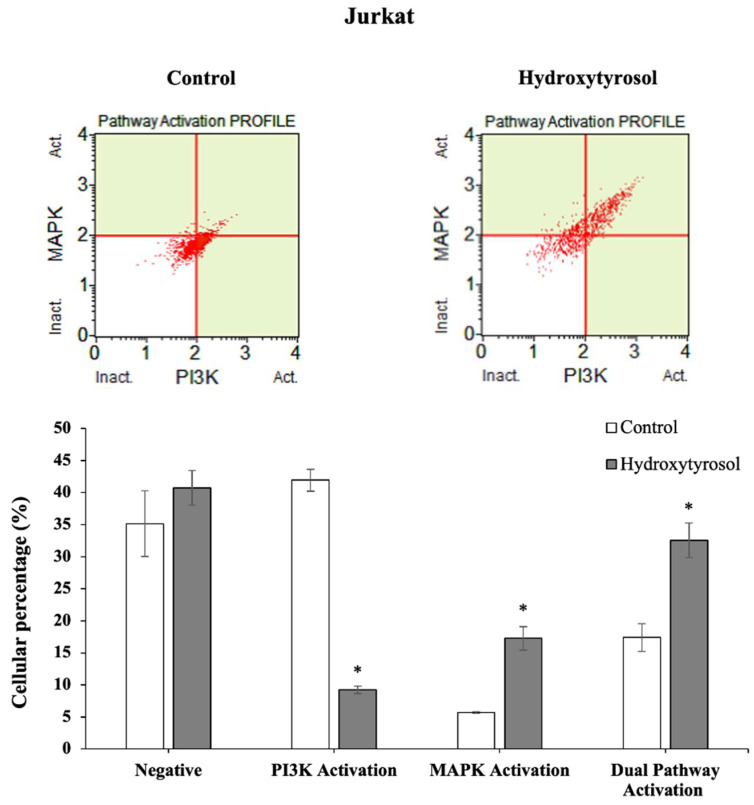
AKT/PI3K and ERK_1/2_/MAPK dual pathway determination was performed according to the MuseTM PI3K/MAPK Dual Pathway activation kit. Graphs correspond to Jurkat cells. Treatments included cells not treated and cells incubated with IC_50_ of HT for 24 h. Top: dot plots show a representative experiment of the treatments. Bottom: represents, using bar diagrams, the percentages of non-activated cells and cells activated with PI3K, MAPK and Dual pathway. Values are expressed as mean ± SEM (*n* = 12). The asterisk indicates significant differences in the different activated pathways between control and treated cells (*p* < 0.001). ERK1/2, Extracellular regulated kinase ½ (Protein-serine/threonine kinases); MAPK, Mitogen-activated protein kinase; AKT/PI3K, Protein kinase B/Phosphoinositide 3-kinase.

The abovementioned results indicate that treatment with HT showed a clear and significant pro-apoptotic effect in these blood cancer lines and therefore it was necessary to understand the nature of the molecular mechanism responsible for the said anticancer activity. For this, whether or not this effect occurs through the dual PI3K/MAPK signaling pathway was studied. The results are shown in Figure 7.

HT treatment produced a significant decrease of 77.9% in cells that only activated the AKT/PI3K pathway, while the percentage of cells activated in the ERK1/2/MAPK pathway was 204%. As result, an increase of 87.2% was observed in cells that activated the dual pathway (MAPK and PI3K).

### 2.7. Hydroxytyrosol Reduces Inflammation Levels in Raw264.7 Cells

**Figure 8:**
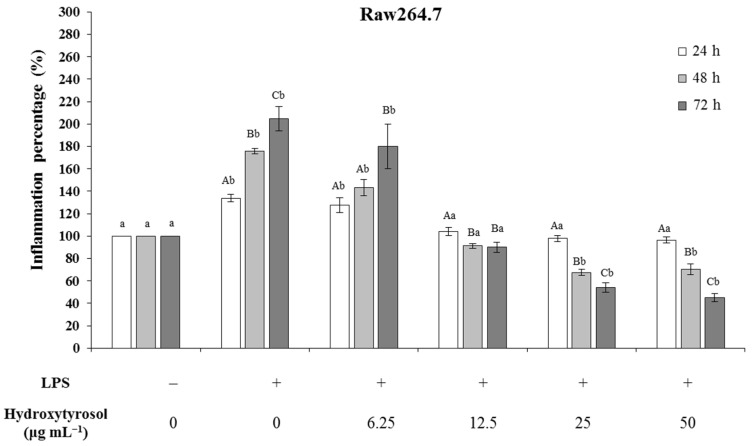
Effect of hydroxytyrosol on the percentages of inflammation measured in terms of nitric oxide (NO) production in Raw264.7 cells when these cells were previously stimulated with LPS (0.1 μg·mL^−1^) and cultured with different concentrations of hydroxytyrosol (0–50 μg·mL^−1^) at 24, 48 and 72 h. The percentage of inflammation of the negative control is represented as 100%. The test was performed in triplicate (*n* = 3) for each experimental condition. Capital letters indicate statistically significant differences (*p* < 0.05) in the same experimental condition (e.g., LPS (+) & HT (6.65 μg·mL^−1^)) between the different times studied (24, 48 and 72 h). Lowercase letters indicate significant differences (*p* < 0.05) between the different experimental conditions for a specific time (e.g., 24 h). LPS, Lipopolysaccharide.

Cells from the Raw264.7 line (mouse leukemic monocyte-macrophage) were used to study the anti-inflammatory potential of HT. A first approach to this study was carried out by measuring the concentration of nitric oxide (NO), in the form of nitrites in the extracellular medium. Figure 8 shows the percentage of inflammation, represented as the relationship between the concentration of NO for each experimental condition and the concentration of the negative control (0 μg·mL^−1^ lipopolysaccharide (LPS) and 0 μg·mL^−1^ HT), a situation that is considered similar to 0% inflammation. The situation in which LPS (0.1 μg·mL^−1^) is administered without the presence of HT and is recognized as a positive control (100% inflammation).

The results revealed that in this cell line, the LPS (positive control) gradually and significantly increased the percentage of inflammation in the three times analyzed (34.7% at 24 h, 74.9% at 48 h and 104.7% at 72 h). To observe the possible anti-inflammatory capacity of HT, different concentrations of this compound were used, which ranged from 6.25–50 μg·mL^−1^. The inflammation values obtained at 24 h demonstrate that low doses of HT (6.25 μg·mL^−1^) did not counteract the inflammation produced by LPS. However, from the 12.5 μg·mL^−1^ dose, the inflammation decreased to the levels of the baseline inflammation generated by the negative control without LPS.

After 48h of treatment, the inflammation gradually decreased, even at low concentrations. In this way, from 6.25 μg·mL^−1^, NO production was reduced by half, finding no differences with the IC_50_ (25 μg·mL^−1^). At 72h, the anti-inflammatory effect of HT followed the same pattern as at 48 h, although the differences were much more marked, reaching levels of inhibition of inflammation of 60% compared to the negative control and more than 80% compared to the positive control (Figure 8).

**Figure 9:**
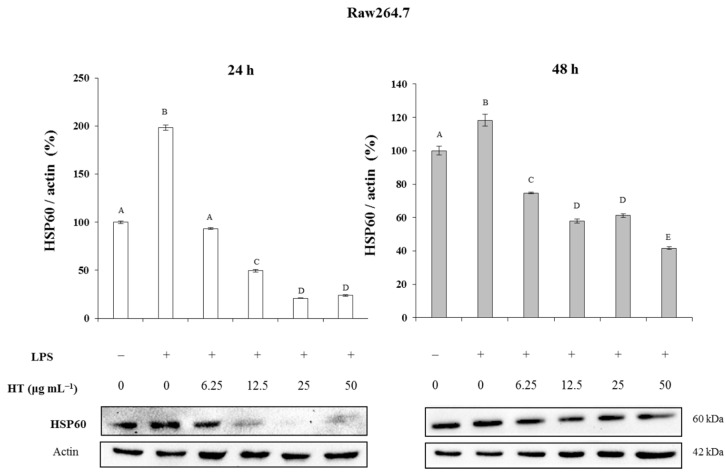
Effect of hydroxytyrosol (HT) on the expression levels of HSP60, a marker protein of the inflammation process in Raw264.7 cells previously subjected to inflammation caused by LPS (0.1 μg·mL^−1^). Quantification of protein levels by densitometric analysis is shown in bar graphs. Results are means ± SEM (*n* = 9) and are expressed as percentage of expression compared to actin levels in Western-blot analysis. Different capital letters indicate the existence of significant differences (from *p* < 0.01 to *p* < 0.001) in the different experimental conditions. HSP60, Heat shock proteins (Chaperon) of 60 kDa; LPS, Lipopolysaccharide.

Finally, and with the aim of understanding some of the molecular aspects responsible for this inflammatory/anti-inflammatory process, the expression levels of an inflammation marker protein, HSP60, a member of a family of proteins that play an essential role, were studied in the recovery of denatured proteins under stress conditions and in the synthesis of proteins during the processes of cell growth, inflammation and survival. The results obtained under the different experimental conditions are shown in Figure 9.

These results showed that when inflammation is induced by the administration of LPS, the expression of HSP60, at 24 h, significantly increased by 103% with respect to the negative control, while at the same time, this increase at 48 h was only 22.6% (*p* < 0.05). However, when the cells previously treated with LPS were administered with various concentrations (6.25, 12.5, 25 and 50 μg·mL^−1^) of HT, the expression levels of the HSP60 protein decreased significantly both at 24 and 48 h, reaching reductions of 71.2% and 85.1% at 24 h and of 85.0% and 65.5% at 48 h compared with the negative and positive controls, respectively (Figure 9).

**Figure 10:**
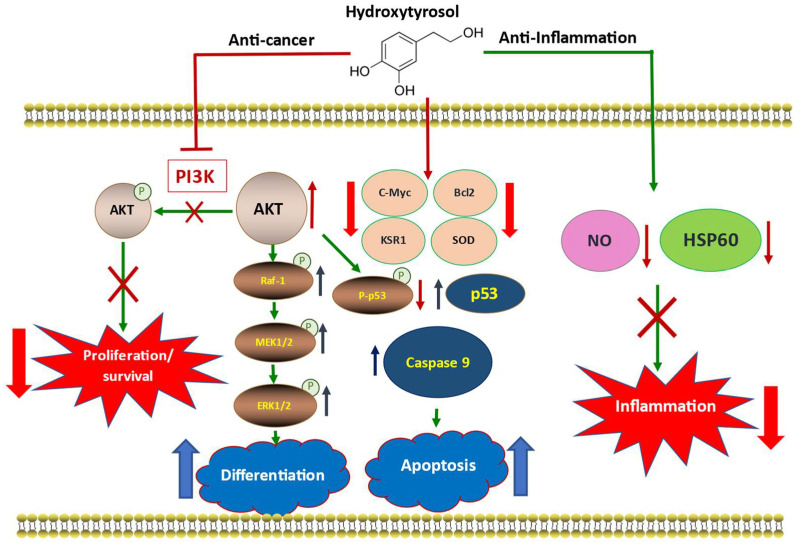
Schematic representation of the main molecular events responsible for the anti-cancer and anti-inflammatory capacity of hydroxytyrosol. The incorporation of HT into the cell triggers a profound inhibition of PI3K activity within the AKT/PI3K signaling pathway. This inhibition allows AKT to avoid phosphorylation, keeping its activity intact, generating a series of molecular changes that lead to a decrease in the proliferation and survival of cancer cells together with an increase in cell differentiation and apoptosis. The activity of AKT facilitates the development of the MAPK pathway through MEK_1/2_/ERK_1/2_, which together with a decrease in the expression levels of c-Myc and KSR1 favors cell differentiation. In addition, HT reduced the levels of the inactive form of p53, increasing the levels of the active form, as well as those of caspase 9 and induced a decrease in the anti-apoptotic protein Bcl-2, which led to a significant increase in the recognition of apoptosis of cancer cells. In parallel, the significant reduction in inflammation caused by the administration of LPS together with a drastic decrease in the expression levels of HSP60 and SOD after treatment with HT allows us to recognize the important anti-inflammatory activity of this natural polyphenol.

Figure 10 summarizes the main molecular pathways that explain the different effects of this phenolic compound.

## 3. Discussion

In recent years, numerous publications revealed the antitumor capacity of HT due to its potential to decrease not only oxidative stress, but also the high proliferation, migration and cell invasion characteristics of cancer. Furthermore, this compound has been reported to decrease inflammation, which is related to angiogenesis that occurs in the carcinogenic processes [41]. In this context, the present work aimed to determine the anticancer capacity of HT in acute type T and acute promyelocytic leukemia lines, evaluating its antiproliferative and proapoptotic effect, as well as its anti-inflammatory effect with a murine macrophage cell line.

Regarding the cytotoxic capacity of HT, the data obtained in this study are consistent with those previously published in the literature, although in other types of cancers. The results obtained are the first data available on cytotoxicity in Jurkat cells (IC_50_ at 24 h, 27.32 μg·mL^−1^) but not in HL60 cells (IC_50_ at 72 h, 23.25 μg·mL^−1^). In this way, different studies described chronic myeloid leukemia K572 [5], colon adenocarcinoma HT29 [35] and breast cancer MCF7 cell lines [34,42], showing values at 24h of IC_50_ 23.12 μg·mL^−1^, 115.6 μg·mL^−1^ and 7.9 μg·mL^−1^, respectively. However, in HL60 cells from acute myeloid leukemia, the IC_50_ obtained in this study at 24 h does not correspond to that obtained in the study carried out by Rosignoli et al. [43] and Fabiani et al. [35]. In those studies, using HT with a purity of 98%, the IC_50_ at 24 and 72 h was determined and the results showed a value of 10.33 μg·mL^−1^ and 7.71 μg·mL^−1^, respectively.

A possible reason for this is the difference in the purity of used HT. In Raw264.7 cells, the cytotoxicity of HT was described by measuring the activity of lactate dehydrogenase (LDH), producing higher values than those found in this study (25 μg·mL^−1^) [44]. Given the results, it would also be interesting to compare this effect of HT on normal T lymphocytes or related blood cells, to study its possible selectivity against cancer cells, which is an undescribed effect.

To confirm our hypothesis and determine the anticancer effect of HT, cell cycle and apoptosis assays were carried out. During the G_0_/G_1_ phase, cell preparation for cell cycle initiation and cell growth began with protein and RNA synthesis, followed by DNA replication during the S phase [45,46]. It is known that the G_1_ checkpoint controls DNA status and cellular activities, also controlling DNA replication in the next phase of synthesis (S phase) [47].

The results of the cell cycle assay in both Jurkat and HL60 cell lines showed that HT induced cell arrest in the G_0_/G_1_ phase, with the consequent decrease in cell proliferation. The same effect on the cell cycle, in response to HT, was demonstrated in different cell lines such as HL60, which showed an arrest of 20% in G_0_/G_1_ phase when an HT treatment at concentrations of its IC_50_ to 24 h (7.7 µg·mL^−1^) [33,35] was applied. López de las Hazas et al. [36] showed that in the CaCo-2 cell line, HT also induced this arrest of 20% at similar doses to those used in our cell lines, but within a shorter time period (8 h).

During the G1 phase, different regulatory genes cause a marked attenuation in cell proliferation by modulating the expression of the suppressor genes p53 and p21, which in turn regulate both cyclins D1 and CDK2 [48]. The tumor suppressor protein p53 is one of the transcription factors that regulates the cell cycle and apoptosis. Normally, in the case of DNA damage and excessive cellular stress, its expression levels and activation increase in order to stop the cell cycle, allowing DNA repair or entry into apoptosis [48]. However, p53 is mutated or silenced in 50–55% of human cancers [49] and, therefore, it is one of the most widely studied targets in the search for new anticancer compounds. In the present study, in the two cell lines used, p53 was not functional since it presented a nonsense mutation according to the Cancer Cell Line Encyclopedia of the Broad Institute. HT treatment in Jurkat and HL60 cells resulted in a decrease in p53 expression. As this protein is mutated and could cause greater proliferation, its lower expression could be due to the antiproliferative effect of HT.

The transcription factor c-Myc is involved in cell cycle regulation and progression. c-Myc is deregulated in almost half of human liquid tumors, such as leukemia, where it is overexpressed. This is because it induces the expression of several positive cell cycle regulators, such as cyclin D/CDK4,6 and cyclin E/CDK2, while blocking cell cycle inhibitors, such as the activation of p21 by p53 [48,50]. The expression of c-Myc in Jurkat and HL60 decreased in the presence of HT in the control, which could be related to the expression of p53 observed in this study. In this way, the decrease in c-Myc would increase the activation of p21 by p53 and the arrest of the cell cycle in the observed phase. This fact is also consistent with other results previously described for HL60 cell line [33].

For its part, KSR1 is involved in the activation of the MAPK pathway, also related to cell proliferation. KSR1 forms a complex with Ras-Raf, MEK and ERK, facilitating the consecutive phosphorylation of ERK by MEK and the signal transduction of this pathway [51]. In our work, we found that KSR1 expression decreased when cells were treated with HT, which could decrease the activation of this proliferation pathway. In addition, this pathway is involved in the induction of c-Myc expression, so the decreased expression of KSR1 would be related to the observed lower expression of c-Myc.

HT has a high pro-apoptotic activity which has already been described in other cell lines such as CF7 [52]. Similarly, in Jurkat and HL60, HT induced apoptosis in a very significant way. Based on these results, the observed decrease in non-functional p53 (P-p53) could be related to the induction of apoptosis, since this transcription factor mediates the cell apoptosis process by activating the death receptor pathway or the apoptotic intrinsic pathway through Apaf1, Noxa and Bax [14,15,16,17,19,20].

The mitochondrial pathway is mainly regulated by pro-apoptotic proteins of the Bcl-2 family, such as Bax, which acts by interacting with p53, releasing cytochrome c [14,15,16,17,19]. Furthermore, since no relevant changes were observed in the expression of Bcl-2, we studied if there was an increase in caspase 9, a marker of the mentioned mitochondrial pathway. Its activation from procaspase 9 to caspase 9, in the apoptosome, initiates a cell signaling that makes the cells unviable [14,15,16,17,19,20,53]. In this case, the expression of caspase 9 was higher than the control, so it was assumed that the intrinsic pathway apoptosis was highly induced by treatment with HT. This corresponds to published articles where the effect of this compound in the HL60 line was observed, as well as how the expression levels of various proteins were modified, resulting in an increase in caspase 9, inducing cell apoptosis [54].

The PI3K/AKT pathway is known as one of the most important signaling pathways involved in cellular processes such as cell growth, cell proliferation, programmed cell death, and cytoskeletal rearrangement [55,56]. Protein kinase B, also named AKT, is the main downstream target of the PI3K/AKT pathway. AKT directly phosphorylates two apoptotic proteins, caspase 9 and BAD, inhibiting their activity and therefore promoting cell survival [57]. In addition to its interaction with the effectors of the Bcl-2 family, the survival signal to cells is transferred by phospho-AKT (p-AKT) [58].

The results of the present study showed that HT significantly decreased the PI3K/AKT pathway in Jurkat cells. In contrast, the ERK_1/2_/MAPK levels were increased. This up-regulation in the MAPK pathway was a consequence of the down-regulation in PI3K, since the PI3K and Ras pathways can cross by crosstalk between their downstream effectors [59]. This effect might explain both the increase in caspase 9 levels and the decrease in Bcl-2 levels caused by HT and therefore the higher apoptosis levels. In other studies of hepatocarcinoma (HepG2, Huh-7, Hep3B and Sk-Hep-1 cell lines) that used triterpenoids, such as uvaol, ursolic acid or platycodin D, the results also showed an inhibition of the PI3K/AKT signaling pathway [26,60,61]. These results support the hypothesis that HT-induced apoptosis is mediated by the modulation of the PI3K/AKT signaling pathway in Jurkat cells.

In the cancer process, ROS levels usually increase due to the generation of a pro-oxidant environment maintained over time. This situation of oxidative stress, generally induced by an exacerbated production of free radicals or by an imbalance in endogenous cellular antioxidant systems, can lead to the production of DNA damage, an initial step in neoplastic development [62]. For this reason, controlling ROS levels inside a cancer cell could be a good strategy to mitigate the genetic material damage or even prevent this. Polyphenols, such as HT, are characterized by having antioxidant activity.

In spite of the antioxidant effects of HT at low doses (those present in olive oil), it has been proven that at high concentrations, HT can act as an enhancer in the production of hydrogen peroxide, acidifying the medium and subjecting cells to stress, which could lead to the induction of apoptosis [63]. A study in several cancer cell lines may explain this process [64]. In this study, HT was incubated together with other compounds, known for their antioxidant potential, such as ascorbate or α-tocopherol and it was observed that in the presence of these compounds, the HT lost apoptotic potential, whereas in their absence apoptosis increased.

In this way, it was deduced that apoptosis induced by HT is due to an overproduction of ROS. This is consistent with the results obtained in Jurkat and HL60 cells, where HT induced higher ROS levels and subsequent apoptosis. The analysis of SOD protein expression, which catalyzes the dismutation of the superoxide anion to hydrogen peroxide and oxygen in HL60 cell line showed a reduction in its expression after HT treatment [65]. When this decrease occurs, the accumulation of ROS generates oxidative damage and leads to the possible induction of apoptosis [65].

Finally, regarding the anti-inflammatory capacity of HT, a reduction in NO production by Raw264.7 was observed of up to 50% at 24 h and up to 100% at 72 h with a concentration of 12.5 μg·mL^−1^ compared to the positive control. This same result had already been demonstrated in the same cell line [44], where it was observed that this capacity was related to the suppression of NFκβ signaling and the decrease in the LPS-mediated expression of iNOS, COX-2, and TNFα [44].

This effect was also demonstrated in the THP-1 cell line [39,40]. HT produced a decrease in HSP60 expression in Raw264.7. As the HSP60 protein has been described as a pro-inflammatory cytokine that induces the secretion of IL-6 and TNFα and NO by macrophages, our result agrees with those of Yonezawa et al., [44], which explains the decrease in inflammation that we highlighted above.

Considering the results reported and the evidences of HT’s effect on the cell cycle and apoptosis through the PI3K/AKT pathway, an in silico analysis of how HT binds to its targets by protein–ligand docking could be performed, as well as a prediction of its possible binding targets. This article provides results confirming the effect of HT extract on standardized hematological cancer cell lines, so our next objective is to use primary cultures from different patients with hematological malignancies and to test whether the effect of HT on the proliferative and pro-inflammatory behavior of these cell types is maintained. Its effect on healthy blood progenitor cells will also be evaluated.

## 4. Materials and Methods

### 4.1. Cell Culture

Three cell lines were used in this study, human T lymphocyte cell line Jurkat (acute lymphoid leukemia, ALL), human neutrophilic promyelocyte cell line HL60 (acute promyelocytic leukemia, APL), which were used as models for cancer tests, and murine leukemic monocyte-macrophage cell line Raw264.7 used as a model to study the anti-inflammatory effects. All three cell lines were provided by the Scientific Instrumentation Centre of the University of Granada. All cells were cultured in a medium composed of RPMI-1640 High Glucose (Sigma-Aldrich^®,^ St. Louis, MO, USA) supplemented with 10% heat-inactivated fetal bovine serum (FBS; Sigma-Aldrich^®^ St. Louis, MO, USA) and 1% streptomycin/penicillin. The cells were maintained in a humidified atmosphere with 5% CO_2_ at 37 °C.

### 4.2. Natural Extract

The HT, 3,4-dihydroxyphenylethanol (DOPET), (Figure 1) was supplied by the company Extractos y Derivados SL (Polígono Industrial de Escúzar, Granada, Spain), as a natural extract of the olive pulp with a richness of 70% of phenolic compounds of which 70% corresponded to HT for which the exact composition is shown in Table 1, and whose extraction process is protected under a patent. At the beginning, a stock solution of the extract dissolved in DMSO with a final concentration of 4 mg·mL^−1^ of HT was prepared, taking into account the richness and density of the extract. Later, it was diluted in culture medium until reaching the concentrations required for each experimental test.

### 4.3. Cytotoxicity Assays

The effect of treatment with HT on proliferation in Jurkat and HL60 cells was measured using the fluorometric method of resazurin, which is based on the mitochondrial reduction of viable cells in resorufin, which could be measured in a spectrofluorometer equipped with a 535 nm excitation and a 595 nm emission filter. In contrast, in Raw264.7 it was determined using 3-(4,5-dimethylthiazol-2-yl)-2,5-diphenyltetrazolium bromide (MTT) assay, based on the ability of live cells to cleave the tetrazolium ring, thus producing formazan, which absorbs at 570 nm.

Then, 3 × 10^5^ Jurkat cells were grown on 96-well plates and incubated with HT (0–100 μg mL^−1^). The plates were incubated at 12 and 24 h. In the case of HL60 (5 × 10^5^ cells) and Raw264.7 (2 × 10^5^ cells), the plates were incubated with HT (0–50 µg·mL^−1^ for HL60 and 0–140 µg·mL^−1^ for Raw264.7) at different times, 24, 48 and 72 h. Depending on this, different numbers of cells were grown to avoid confluence as follows: for 24 h, 20,000 cells per well; for 48 h, 17,000 cells; and for 72 h 14,000 cells.

After incubation, 20 μL of resazurin solution (1 mM) or 20 μL of MTT solution (0.5 mg·mL^−1^) was added to each well. After 2 h incubation, 50 μL of SDS 3% was added or the cells were washed twice with PBS and the formazan was resuspended in 200 μL DMSO. Relative cell viability was measured by fluorescence at 535/590 nm or by absorbance at 570 nm on an ELISA plate reader (Synergy HTX multi-mode reader, Biotek^®^ Winooski, VT, USA). The tests were carried out in triplicate.

Results were expressed as a percentage of live cells with respect to the control, which is considered 100%, and errors were calculated as the standard deviation of mean. The concentrations that caused 20%, 50% and 80% inhibition of cell viability (IC_20_, IC_50_ and IC_80_) were calculated according to the method used by Bonel-Pérez et al. [26].

### 4.4. Cell Cycle Analysis

The 3 × 10^5^ Jurkat cells were grown on a 6-well plate and incubated with IC_50_ values of HT for 24 h. After this, the cells were centrifuged at 469× *g* for 5 min and the pellet was resuspended in 500 µL of TBS (10 mM Tris, 150 mM NaCl, pH 7.4), and 25 µL of PI (1 mg·mL^−1^) and 500 µL of Vindelov/PI buffer (10 mM Tris, 10 mM NaCl, 10 µg mL^−1^ RNase, 50 µg·mL^−1^ PI, 0.01% Igepal) were added until a final concentration of 100 µg·mL^−1^ of PI. After incubating for 1 h at 4 °C, cells were analyzed by flow cytometry at 488 nm with an Epics XL flow cytometer (Coulter Corporation, Hialeah, FL, USA). The percentage of cells in each phase was calculated using Multicycle program. The experiments were carried out at least in triplicate.

We grew 5 × 10^5^ HL60 cells per well and incubated them with the IC_50_ value at 72 h. After incubation, the Muse™ Cell Cycle Kit (EMD Millipore Corporation, Hayward, CA, USA) was used following the specification of the manufacturer and the samples were analyzed in the Muse™ Cell Analyzer. The experiments were carried out at least in triplicate.

### 4.5. Apoptosis Assay

Phosphatidylserine on the outside of the apoptotic cells was determined by an annexin V-FITC apoptosis kit (Calbiochem, San Diego, CA, USA). We grew 3 × 10^5^ Jurkat cells on a 6-well plate and incubated them with IC_50_ values of HT for 24 h. The cells were centrifuged and the pellet was resuspended in 300 µL of a 1× binding solution (10 mM HEPES/NaOH pH 7.4; 140 mM NaCl, 2 mM CaCl_2_). It was centrifuged again and the pellet was resuspended in 95 µL of the same solution. Then, 3 µL of Annexin V-FITC was added and incubated for 30 min at RT and in darkness. Finally, 300 µL of the 1× binding solution was added and, 5 min before reading, 5 µL of PI (1 mg·mL^−1^). The analysis was made at 480 nm by flow cytometry. These experiments were carried out at least in triplicate.

Apoptosis death in HL60 was performed using Muse^®^ Annexin V Dead Cell Kit (EMD Millipore Corporation, Hayward, CA, USA) following the recommendations of the manufacturer. The conditions used were as follows: 5 × 10^5^ HL60 cells per well were grown and incubated with the IC_50_ value at 72 h. These experiments were carried out at least in triplicate.

A positive control was carried out for both cell lines, incubating cells with staurosporine (1 μg·mL^−1^), a nonspecific inhibitor of kinase proteins isolated from the *Streptomyces staurosporeus* species, which revealed the ability to induce the apoptotic pathway in a large number of tumor lines [26].

### 4.6. Measurement of ROS Production

For this purpose, the Muse^®^ Oxidative Stress kit was used, which is based on the use of dihydroethidium (DHE), a reagent with the ability of crossing the cell membrane and interacting with superoxide anions, a type of ROS. In this process, DHE oxidized and formed the DNA intercalating agent ethidium bromide, detectable by its fluorescence emission. This allowed for two cell populations to be distinguished, those that express remarkable levels of ROS, ROS (+) and those that do not ROS (−). Before following the Muse™ Cell Cycle Kit protocol, Jurkat and HL60 cells were prepared as in the cell cycle study.

### 4.7. Preparation of Cytosolic Extracts

Triplicates of two different populations of each cell line, Jurkat and HL60, were incubated with IC_50_ concentrations of HT for 24 h and 72 h, respectively. Triplicates of Raw264.7 cells were incubated with IC_50_ and half of IC_50_ for 24 and 48 h. In addition, they were incubated with and without LPS (0.1 µg·mL^−1^).

Cells were centrifuged at 469g for 5 min and recollected in RIPA buffer (50 mM Tris-HCl pH 7.5; 150 mM NaCl to keep the osmotic pressure near a physiological level; 1% non-ionic detergent NP-40; 0.5% deoxycholic acid and 0.1% SDS as anionic detergents; 0.2 mM protease inhibitor PMSF; 7 mM Na_3_VO_4_ as phosphatase inhibitor and Thermo Scientific^®^ PierceTM inhibitor cocktail, Rockford, IL, USA). Immediately, cells were sonicated on ice for 5 min and maintained in moderate shaking at 4 °C for 1 h. Every 15 min, the samples were moderately shaken in a vortex. Lysates were centrifuged at 10,000× *g* at 4 °C for 15 min. The supernatants were used for Western blot assays, and the protein concentration was measured using the Bradford method (Bio-Rad^®^, Munich, Germany).

### 4.8. Western Blotting

Polyacrylamide gel electrophoresis under denaturing conditions (SDS-PAGE) was performed in a Mini-Protean II electrophoresis system (Bio-Rad, Richmond, VA, USA). The cell extracts were mixed with a charge buffer that contained 62.5 mM Tris-HCl at pH 6.8, 20 g·L^−1^  SDS, 100 mL·L^−1^  glycerol, 25 g·L^−1^  β-mercaptoethanol and 0.045 mM bromophenol blue and then heated for 5 min at 100 °C. Polypeptides were separated on a 10% or 12% SDS-PAGE and subsequently transferred to polyvinylidene fluoride membranes with a semi dry electroblotting system at 1.5 mA·cm^−2^ for 45 min in a medium containing 25 mM Tris-HCl, 192 mM glycine, 200  mL·L^−1^ methanol, and 1  g·L^−1^ SDS.

Blots were blocked for 1h at RT with a Tris buffered solution (TBS) that contained 25 mM Tris-HCl, 100 mM NaCl, 2.5 mM KCl, pH 7.6, 1 mL·L^−1^ Tween 20, and 15 g·L^−1^ bovine serum albumin (BSA) at pH 7.6. Membranes were washed with TBS containing 1 mL·L^−1^ Tween 20 (TBS-T) for 15 min and later incubated with the following specific primary antibodies: anti-Bcl-2 antibody 1:1000 (Santa Cruz Biotechnology, Dallas, TX, USA (SCBT, sc-7382)), anti-caspase 9 antibody 1:1000 (SCBT, sc-7885), anti-c-Myc antibody 1:1000 (Cell Signaling, D3N8F), anti-HSP-60 antibody 1:250 (SCBT, sc-1722), anti-KSR1 antibody (1:1000) (SCBT, sc-9317), anti-p53 antibody 1:500 (SCBT, sc-6243), anti-SOD antibody 1:250 (SCBT, sc-101523) and anti-α-actin 1:1000 (Sigma^®^, St. Louis, MO, USA, A2668). Following three washes with TBS-T containing 10 g·L^−1^ BSA (TBS-T-BSA) for 10 min, membranes were incubated with HRP conjugated goat anti-rabbit antibody IgG, anti-goat IgG or anti-mouse IgG (1:5000).

These secondary antibodies have the enzyme horseradish peroxidase (HRP) coupled, which permits chemiluminescence due to its ability to oxidize the luminol solution (ECL-plus Western-blot detection system, GE Healthcare, Chicago, IL, USA). The reaction produced was captured inside a ChemiDoc Imaging System (Bio-Rad^®^ Munich, Germany). The program used to analyze the images obtained was Image Lab Software (Bio-Rad^®^ Munich, Germany). The expression of each protein was normalized referring to actin levels and the results were expressed as percentage of expression, taking the negative control as 100%. The expression obtained in each protein was analyzed through the mentioned program, at least in triplicate.

### 4.9. PI3K/MAPK Dual Pathway Activation Assay

For this assay, the Muse^®^ PI3K/MAPK Dual Pathway activation kit purchased from Millipore (Billerica, MA, USA) was used to examine PI3K and MAPK signaling pathways simultaneously using the Muse Cell Analyzer (Merck-Millipore^®^, Burlington, MA, USA). The protocol followed was carried out according to the manufacturer’s instructions [26]. The samples (quadruplicates of three different populations of Jurkat cells) were analyzed in the above-mentioned MuseTM Cell Analyzer.

### 4.10. Study of Inflammation Using the Griess Method

The method used to detect nitrites was the Griess method based on the use of the following two reagents under acidic conditions: 1% sulfanilamide in 5% H_3_PO_4_ and 0.1% NED (N-1-naphthylethylene diamine). If those compounds reacted with NO, a colored azole compound was produced, whose absorption peak was 548 nm.

The 2 × 10^5^ cells per well of Raw264.7 were seeded in 96-well plates. Different conditions were used at least in triplicate: negative control (cells without LPS or HT), positive control (cells with 0.1 µg·mL^−1^ of LPS) and cells treated with different proportions of IC_50_ at 24 h: 6.25, 12.5, 25 and 50 µg·mL^−1^, all of them previously stimulated with LPS (0.1 µg·mL^−1^). In addition, two incubation times were applied, namely 24 and 48 h.

After that, 150 µL of the supernatant was placed in each well of the other plate and 25 µL of sulfanilamide and 25 µL of NED were added, respectively. It was incubated at RT for 20 min and the absorbance at 548 nm was measured in the aforementioned plate reader. The values obtained from the nitrite concentration were used to calculate the percentage of inflammation of the cells, taking 100% as the value given by the negative control cells.

### 4.11. Statistical Analysis

The results were expressed as the mean ± standard error of the mean (SEM). Likewise, the effects of different treatments on the studied parameters were analyzed independently using one-way analysis of variance (one-way ANOVA), followed by Tukey’s HSD test. The differences were considered significant at *p* values less than 0.05, (*p* < 0.05). Statistical analyses were performed using the IBM SPSS Statistics (IBM^®^ Armonk, NY, USA) software (version 25).

## 5. Conclusions

Both the glucoside oleuropein (a phenolic component of the leaves and pulp of olives of the *Olea europaea* variety), as well as its major metabolite, hydroxytyrosol, have generated great interest among researchers in recent years. So, there is a common objective of searching for new pharmacological properties of this compound and potential new applications, in addition to those already known such as its antioxidant, immunostimulant and cardioprotective effects.

This study demonstrated and supported that hydroxytyrosol has a potential antiproliferative effect by affecting cell arrest in the G_0_/G_1_ phase of the cell cycle, a significant anticancer effect due to an increase in cell apoptosis through the activation of the main molecular markers of the intrinsic signaling pathway, as well as an increase in cellular oxidative stress due to an increase in ROS levels, in two human leukemia cell lines, HL60 and Jurkat. As a complement to all this, it should also be noted that hydroxytyrosol showed a high anti-inflammatory effect in the Raw264.7 cell line, since it caused a significant decrease in HSP60 levels after incubation with LPS. In our opinion, as demonstrated in the present study, HT could be regarded as a potential source of natural anticancer and anti-inflammatory effects.

## Figures and Tables

**Table 1:** Composition of the extract OLIVESAN^®^ 48% Syrup (olive extract rich in phenols).

Olive Extract Composition			
Class	Ingredients	*w*/*w*	Total %*w*/*w*
Olive Fruit Phenols Acid	Hydroxytyrosol	48.00	70.00
	Hydroxytyrosol Acetate	0.25	
	Tyrosol	8.25	
	Oleuropein (aglycone)	7.50	
	Vainillic Acid	2.00	
	Coumaric Acid	0.60	
	Caffeic Acid	0.80	
	Catechol	0.75	
	Luteolin	0.25	
	DHPG (*1*)	0.60	
	Other phenols (*2*)	1.00	
Olive oil Fats	Saturated fats	0.05	25.30
	Unsaturated fats	25.25	
Proteins	Proteins	0.10	0.10
Carbohydrates	Sugars (mannitol)	3.00	3.20
	Others	0.20	
Salt	Salt	0.30	0.30
Organic acids	Organic acids (*3*)	0.55	0.55
Water	Water	0.55	0.55
Total		100.00	100.00

Physical-chemical characterization: Physical state: Liquid with high density and viscosity. Color: Dark olive; Smell: Slightly sweet; PH value: 6.0–7.0; Melting temperature: −11 °C; Boiling point: 105 °C; Flammability limit: 310 °C; Autoignition temperature: 350 °C; Explosion limit: Not stated; Vapor pressure: 16.5 mm Hg; Density: 1.2034 kg·L^−1^; Viscosity: 600 cP @ 25 °C (approx.); Water solubility: Soluble in water. Notes: (*1*) Dihydroxyphenylglycine, (*2*) Other phenols: olivil, azelaic acid, etc. (*3*) Organic acids: citric acid, acetic acid. Manufacturer: Extractos y Derivados, S.L. (Polígono Industrial de Escúzar, Granada, Spain). Date of analysis: March 2019.

## Data Availability

The data presented in this study are available on request from the corresponding author.

## Abbreviations

**Apaf1**, Apoptotic protease activating factor 1; **ATRA**, All-trans-retinoic acid; **Bax**, Pro-apoptotic Bcl-2-associated X protein; **Bcl2**, Anti-apoptotic B-cell lymphoma 2; **CDKs**, Cyclin-dependent kinases; **cMyc**, Protein expressed from cMYC proto-oncogene; **COX-2**, Cyclooxygenase 2; **DHE**, Dihydroethidium reagent; **DMSO**, Dimethyl Sulfoxide reagent; **ERK_1/2_**, Extracellular regulated kinase ½ (Protein-serine/threonine kinases); **Hepes**, 4-(2-hydroxyethyl)-1-piperazineethanesulfonic acid reagent; **HSP60**, Heat shock proteins (Chaperon) of 60 kDa; **HT**, Hydroxytyrosol; **IC_20,50,80_**, Concentration of a drug that gives 20%, half-maximal and 80% responses; **iNOS**, Inducible nitric oxide synthase; **KSR1**, Kinase suppressor of Ras 1; **LPS**, Lipopolysaccharide; **MAPK**, Mitogen-activated protein kinase; **MEK_1/2_**, Mitogen-activated protein ERK Kinase Kinases (Protein-serine/threonine kinases); **MTT**, 3-(4,5-dimethylthiazol-2-yl)-2,5-diphenyltetrazolium bromide reagent; **NED**, N-1-naphthylethylene diamine reagent; **NF-Kβ**, Nuclear factor kappa-light-chain-enhancer of activated βeta cells; **NO**, Nitric oxide; **Noxa**, Phorbol-12-myristate-13-acetate-induced protein 1; **PBS**, Phosphate buffered saline; **PI**, Propidium iodide; **AKT/PI3K**, Protein kinase B/Phosphoinositide 3-kinase; **PKCα**, Protein kinase C alpha; **PKCβ1**, Protein kinase C beta1; **PMSF**, Phenylmethylsulphonyl fluoride reagent; **ROS,** Reactive oxygen species; **RT**, Room temperature; **SDS**, Sodium dodecyl sulfate; **SOD**, Superoxide dismutase; **TBS**, Tris-buffered saline; **TNFα**, Tumor necrosis factor α.
